# PolyI:C Upregulated *CCR5* and Promoted THP-1-Derived Macrophage Chemotaxis via TLR3/JMJD1A Signalling

**DOI:** 10.22074/cellj.2020.6713

**Published:** 2019-12-15

**Authors:** Xiaoxiao Yu, Huayang Wang, Hongjia Shao, Cuijuan Zhang, Xiuli Ju, Jie Yang

**Affiliations:** 1. Department of Paediatrics, Qilu Hospital of Shandong University, Jinan, Shandong, China; 2. Department of Clinical Laboratory, Qilu Hospital of Shandong University, Jinan, Shandong, China; 3. Department of Pathology, Qilu Hospital of Shandong University, Jinan, Shandong, China

**Keywords:** Chemokine Receptor 5, Chemotaxis, Macrophages, Polyinosinic:polycytidylic Acid

## Abstract

**Objective:**

This study aimed to evaluate the specific roles of polyinosinic:polycytidylic acid (polyI:C) in macrophage chemotaxis and reveal the potential regulatory mechanisms related to chemokine receptor 5 (*CCR5*).

**Materials and Methods:**

In this experimental study, THP-1-derived macrophages (THP1-Mφs) induced from THP- 1 monocytes were treated with 25 μg/mL polyI:C. Toll-like receptor 3 (*TLR3*), Jumonji domain-containing protein (JMJD)1A, and *JMJD1C* small interfering RNA (siRNAs) were transfected into THP1-Mφs. Quantitative real-time reverse transcriptase polymerase chain reaction (qRT-PCR) was used to detect the expression levels of *TLR3, CCR5,* 23 Jumonji C domain-containing histone demethylase family members, *JMJD1A*, and *JMJD1C* in THP1-Mφs with different siRNAs transfections. Western blot was performed to detect *JMJD1A*, JMJD1C, H3K9me2, and H3K9me3 expressions. A transwell migration assay was conducted to detect THP1-Mφ chemotaxis toward chemokine ligand 3 (CCL3). A chromatin immunoprecipitation (ChIP) assay was performed to detect H3K9me2-CCR5 complexes in THP1- Mφs.

**Results:**

PolyI:C significantly upregulated *CCR5* in THP1-Mφs and promoted chemotaxis toward *CCL3* (P<0.05); these effects were significantly inhibited by TLR3 siRNA (P<0.01). *JMJD1A* and *JMJD1C* expression was significantly upregulated in polyI:C-stimulated THP1-Mφs, while only *JMJD1A* siRNA decreased *CCR5* expression (P<0.05). *JMJD1A* siRNA significantly increased H3K9me2 expression in THP1-Mφs but not in polyI:C-stimulated THP1-Mφs. The ChIP result revealed that polyI:C significantly downregulated H3K9me2 in the promoter region of *CCR5* in THP1- Mφs.

**Conclusion:**

PolyI:C can enhance THP1-Mφ chemotaxis toward *CCL3* regulated by TLR3/JMJD1A signalling and activate *CCR5* expression by reducing H3K9me2 in the promoter region of *CCR5*.

## Introduction

Acute lung injury (ALI) is an inflammation characterized by the breakdown of the endothelial and epithelial lung barrier (1). Monocyte-derived macrophages are important in the pathogenesis of ALI. Under the pathological conditions of ALI, activated circulating monocytes infiltrate the alveolar space to form alveolar macrophages. Subsequently, alveolar macrophages may secrete several inflammatory mediators, such as cytokines and chemokines, to induce the migration of mature neutrophils and CD4^+^T cells into the alveolar space, thereby prompting an inflammation response that may kill pathogenic microbes (2, 3). A previous study showed that the depletion of circulating monocytes and subsequently recruited alveolar macrophages significantly suppressed ALI in mice (4). Therefore, the function and activity of macrophages are extremely important in the development and prognosis of ALI.

Toll-like receptors (TLRs) are categorized as innate immune sensors, which play an important role in the process of antigen recognition for innate immune cells such as macrophages (5). It has been reported that *TLR3* is upregulated in alveolar macrophages throughout the ALI pathogenesis (6). Chemokines comprise a class of cytokines that act as signalling molecules in the regulation of inflammatory response (7). Chemokine receptors (CCRs) are specific receptors for chemokines that are integral to the recruitment of alveolar macrophages (8). *TLR3* and CCRs participate in ALI-induced inflammatory response through the recognition of pathogen-related molecular processes or the recruitment of macrophages; however, whether a direct regulating mechanism between CCRs and *TLR3* exists in macrophages has not been thoroughly researched.

Histone demethylation is an important form of epigenetic modification that is regulated by Jumonji C domaincontaining histone demethylases (JHDMs) (9). Histone demethylation is involved in the transcriptional repression and activation of target genes, and is closely associated with the inflammatory response of macrophages. It has been reported that Jumonji domain-containing protein 3 (*JMJD3*) influences transcriptional gene expression in lipopolysaccharide (LPS)-activated macrophages, and the regulatory role of *JMJD3* is dependent upon H3K4me3 (10). An H3K27me3 inhibitor reduces LPS-induced proinflammatory cytokine production by macrophages, and this process is regulated by *UTX* and *JMJD3* (11). Moreover, a pervious study reported that high glucose upregulates diverse inflammatory cytokines in macrophages, including *IL-6, IL- 12p40*, and *MIP-1α/β*; this process is closely associated with H3K9 methylation (12). However, the specific role of H3K9 methylation in *TLR3* signalling for macrophage-involved inflammatory responses remains unknown.

Polyinosinic:polycytidylic acid (PolyI:C) is a viral mimetic that mimics inflammatory responses to systemic viral infection (13). In this study, the effects of polyI:C on THP-1-derived macrophage (THP1-Mφ) chemotaxis, as well as potential regulatory mechanisms related to *TLR3* and CCRs, are explored. The aim of this study is to provide new insight into the underlying regulatory mechanisms for macrophage participation in ALI.

## Materials and Methods

### Cell culture and induction of THP-1-derived macrophages (THP1-Mφs)

In this experimental study, human THP-1 monocytes were purchased from the American Type Culture Collection (Manassas, VA, USA) and cultured in RPMI- 1640 medium that contained 10% heat-inactivated foetal bovine serum (FBS, Gibco, USA) and 100 U/ mL penicillin-streptomycin. Cells were maintained in an atmosphere of 5% CO2 at 37˚C. Exponential-phase cells were used in the following assays.

THP-1 monocytes were induced to differentiate into macrophages in vitro. Simply, THP-1 monocytes suspended in RPMI-1640 medium were seeded in 6-well plates at a density of 2×10^5^ cells/mL. Then, 100 ng/mL phorbol-12- myristate acetate (PMA) (Sigma, St. Louis, MO, USA) was added to the THP-1 monocytes. After a 48-hour incubation period, the adherent macrophages were used in the following assays (THP1-Mφs). For polyI:C treatment, THP-1 monocytes were incubated with 100 ng/mL PMA for 6 hours, and then treated with 25 μg/mL polyI:C (R&D Systems, Minneapolis, MN, USA). After 42 hours of incubation, the adherent macrophages were used in the following assays (polyI:C-stimulated THP1-Mφs).

### Quantitative real-time reverse transcriptase polymerase chain reaction

Total RNA was extracted from cells of different groups using TRIzol (Fermentas, Burlington, Ontario, Canada) and reverse-transcribed by RevertAid M-MuLV Reverse Transcriptase (Fermentas, Canada) in accordance with the manufacturer’s instructions. Quantitative real-time reverse transcriptase polymerase chain reaction (qRT-PCR) was performed on a LightCycler 2.0 Instrument (Roche, Germany) using the SYBR Green PCR Kit (TaKaRa, Japan). The relative expression levels of target genes were calculated by 2^-ΔΔCt^, using *GAPDH* as an internal control. The primer sequences are shown in Table 1.

### Flow cytometry

Flow cytometry was performed to detect chemokine receptor 5 (CCR5) expression in THP1-Mφs. Simply, cells were suspended in fresh RPMI-1640 medium and incubated with CCR5-PE antibody (R&D Systems, USA) in the dark for 30 minutes at room temperature. Data were collected using the FACSCalibur flow cytometer (BD Biosciences, San Jose, CA, USA) and analysed with CellQuest software (BD Biosciences).

### siRNA transfection

siRNAs targeting *TLR3*, Jumonji domain-containing protein 1A (*JMJD1A*), and *JMJD1C* were obtained from Shanghai GeneChem Company (Shanghai, China), as follows:

*TLR3* siRNA: 5ˊ-CCUGAGCUGUCAAGCCACUACCUUU-3ʹ
*JMJD1A* siRNA: 5ʹ-GCAAUUGGCUUGUGGUUACUU-3ʹ
*JMJD1C* siRNA: 5ʹ-GCAAUUGGCUUGUGGUUACUU-3ʹ.

After 6 hours of incubation with 100 ng/mL PMA, THP1-Mφs were incubated with specific siRNAs and Lipofectamine 2000 reagent (ThermoFisher, Waltham, MA, USA) for 6 hours. Transfected cells were treated with 25 μg/ mL polyI:C for an additional 42 hours. The efficacy of the *TLR3* transfection was detected using qRT-PCR and flow cytometry as described above, while the efficacy of *JMJD1A* and *JMJD1C* siRNA-mediated gene silencing was monitored using Western blotting.

### Transwell migration assay

THP1-Mφ chemotaxis toward chemokine ligand 3 (*CCL3*) was detected using transwell inserts. Transwell inserts with a pore size of 8 μm were placed into 24-well plates. Cells were suspended in serum-free RPMI-1640 medium and inoculated into the upper chamber at a density of 1×10^5^ cells/mL. RPMI- 1640 medium that contained 100 ng/mL recombinant human CC chemokine ligand 3 (rhCCL3;#270-LD, R&D Systems, USA) and 10% FBS was added into the lower chamber. Following 12 hours of incubation at 37˚C, the non-migrated cells were removed from the upper chamber, and migrated cells in the lower chamber were fixed with methanol and stained with eosin. Five random fields of each well were observed using light microscopy, and the number of migrated cells was counted.

### Chromatin immunoprecipitation assay

The chromatin immunoprecipitation (ChIP) assay was performed to detect H3K9 methylation in THP1-Mφs. After being fixed in 1% formaldehyde, the chromatin was extracted from THP1-Mφs using sonication. Then, the chromatin was immunoprecipitated with H3K9me2 (Abcam, Cambridge, MA, USA) or H3K9me3 antibody (Abcam, USA) pre-bound Protein G-plus Agarose beads, overnight at 4˚C. Precipitated protein-DNA complexes were eluted in Tris-EDTA buffer that contained 2% sodium dodecyl sulfonate (SDS), and the crosslink was reversed through a 16 hour incubation period at 65˚C. The precipitated DNA fragments were analysed by qRT-PCR as described above. The primer sequences of *CCR5-ChIP* are shown in Table 1. qRT-PCR was performed on a LightCycler 2.0 Instrument (Roche, Germany) using TB Green Fast qPCR Mix (Code No. RR430S/A/B, TaKaRa, Japan).

**Table 1:** Sequences of specific primers used in quantitative real-time reverse transcriptase polymerase chain reaction (qRT-PCR)


Gene	Primer sequence (5ˊ-3ˊ)

CCR1	F: CGAAAGCCTACGAGAGTGGAA
	R: CGGACAGCTTTGGATTTCTTCT
CCR2	F: GAGCCATACCTGTAAATGCC
	R: GAGCCCAGAATGGTAATGTG
CCR4	F: CATGAACCCCACGGATATAGCA
	R: CTACTCCCCAAATGCCTTGATG
CCR5	F: TGTCCCCTTCTGGGCTCACTAT
	R: TGGACGACAGCCAGGTACCTA
CCR6	F: TCGCCATTGTACAGGCGACTA
	R: CGCTGCCTTGGGTGTTGTAT
CCR7	F: CCTGGGGAAACCAATGAAAAGC
	R: GAGCATGCCCACTGAAGAAGC
CXCR4	F: TTCCTGCCCACCATGTAGTC
	R: TCGATGCTGATCCCAATGTA
FBXL10	F: CAGTGGGTGGAGGGACTAAA
	R: ACTGAGGTGGAGCTTGGAGA
FBXL11	F: ATAACCAACCGTTCCCACCT
	R: TGCCCAGTCCATCATAATCC
JMJD1A	F: ATGCCCACACAGATCATTCC
	R: CTGCACCAAGAGTCGATTTT
JMJD1B	F: AACTTCCTCAAACCCCCTTG
	R: CCCATCACCATCTCCTTCAC
JMJD1C	F: TCCAGAATCCCAGTCACCAC
	R: CAGCAAATCCCGTAAGGTTG
JMJD2A	F: CAGAGGACCAAGCCATTGAT
	R: ATTGGCTGAACACCGAGAAC
JMJD2B	F: GGGGAGGAAGATGTGAGTGA
	R: CTATGGGTGCCTCCTTCTCA
JMJD2C	F: TGCCTGAGGTTCTGTCCATT
	R: GCTGCTATCTGGCTTGTGGT
JMJD2D	F: AAATATGTACGGGGCAACCA
	R: TACTCAGACCTGGGGGTACG
JMJD3	F: CTGATGCTAAGCGGTGGAAG
	R: TGTTGATGTTGACGGAGCAG
JMJD4	F: ACTGGGTCAATGGCTTCAAC
	R: AGGACCAGGAGCCTCTTCTC
JMJD5	F: ACATCAGCATCCCCGACTAC
	R: AGGGTACAGAGCCCCTGACT
JARID1A	F: TGAACGATGGGAAGAAAAGG
	R: AGCGTAATTGCTGCCACTCT
JARID1B	F: TTGGGATTGAAAAGGAAGCA
	R: CAGCAATTTCCCTTCATTGG
JARID1C	F: CAGGGCTTACTGGAGAATGG
	R: TTCTCATCCAGGGTCACCTC
JARID1D	F: ACTGAACTCCGGGTCCTTCT
	R: GCTTCAGGCACCTCTACACC
JARID2	F: CTGTCTGGAGTGTGCTCTGC
	R: ACGTCCACTGTCGCTCTCTT
UTX	F: CGTGTCGTATCAGCAGGAAA
	R: CACCCCAGTAACCTTCAGGA
HR	F: CAGTCAGCGTCACTCAGCA
	R: CGATCCCAGACACCTAGCA
HSPBAP1	F: AAGCTCAAAGACATGCGGTTA
	R: CAGGCTCTGGTATTTTGTGGA
HIFAN	F: ACAATCCCGACTACGAGAGGT
	R: GCCACTTTCTGATGAGCTTTG
MINA	F: ACTTTGGCTCCTTGGTTGG
	R: CCCGGCTTCAGCATAAAC
PHF2	F: ATCTTTAAGTCCCGGTCGAAG
	R: TTCCTCTTGGCACTCTTTT
PHF8	F: CTGATGATGATGACCCTGCTT
	R: TTCTTCTTTTGGGCCTTCTGT
PHF20	F: ACCCGGCTCCCCAAAGGTGA
	R: CTGCCACTGGTGCTGGGAGC
CCR5-ChIP	F: TGTGGGCTTTTGACTAGATGA
	R: TAGGGGAACGGATGTCTCAG
GAPDH	F: CAACTGGTCGTGGACAACCAT
	R: GCACGGACACTCACAATGTTC


### Western blot

THP1-Mφs were lysed in RIPA buffer. Total proteins were separated by SDS-polyacrylamide gel electrophoresis on 10% polyacrylamide gels and transferred to nitrocellulose membranes (Bio-Rad, Hercules, CA, USA). The membrane was blocked with 5% skim milk in TBST for 2 hours and incubated with special primary antibody (anti-H3K9me2, anti-H3K9me3, Abcam, USA) at 4˚C for 12 hours. After there were washed three times with TBST, the membrane was incubated with horseradish peroxidase-conjugated secondary antibody (Abcam, USA) at 25˚C for 2 hours. Protein bands were visualized with the Image Station IS2000 (Kodak, Rochester, NY, USA).

### Statistical analysis

All experiments were performed in triplicate, and all data are presented as means ± standard deviation. The statistical analysis conducted in this study was performed using SPSS 13.0 (SPSS Inc., Chicago, IL, USA). The Shapiro-Wilk was used to test the normality of the distribution. For the data presenting a normal distribution, the mann-withney (two groups) and kruskal-wallis (more than two groups) were used to compare results among different groups. The Wilcoxon rank-sum test was used for non-normally distributed data. P<0.05 denoted statistically significant results.

## Results

### Polyinosinic:polycytidylic acid upregulated chemokine receptor 5 expression in THP-1-derived macrophages through toll-like receptor 3 signalling

**Fig 1:**
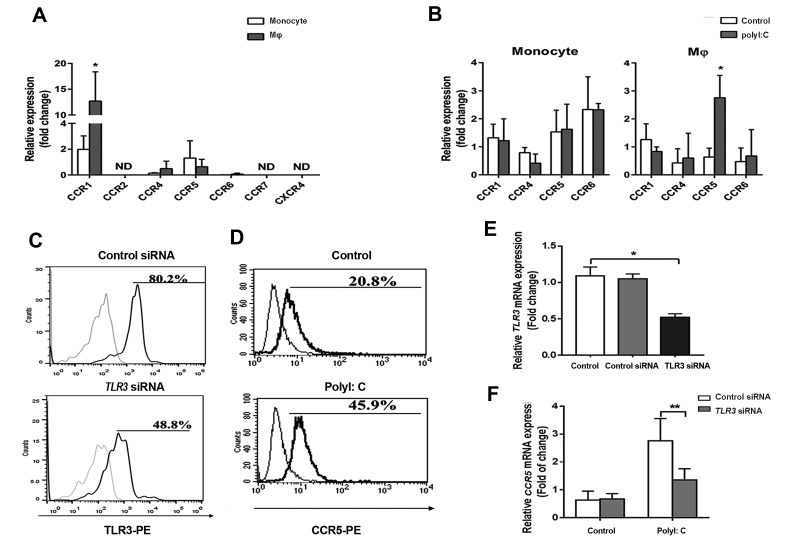
Polyinosinic:polycytidylic acid (PolyI:C) upregulated chemokine receptor 5 (*CCR5*) expression in THP-1-derived macrophages (THP1-Mφs) through toll-like receptor 3 (*TLR3*) signalling. **A.** Expression profile of chemokine receptors in THP-1 monocytes and THP1-Mφs (Mφ) by quantitative real-time reverse transcriptase polymerase chain reaction (qRT-PCR) (fold change at the mRNA level), **B.** *CCR1, CCR4, CCR5,* and *CCR6* expressions in polyI:Cstimulated THP-1 monocytes and THP1-Mφs by qRT-PCR,** C.** CCR5 expression in polyI:C-stimulated THP1-Mφs by flow cytometry, **D.** TLR3 expression in THP1-Mφs with TLR3 siRNA by flow cytometry, **E.** Knockdown efficiency of *TLR3* siRNA by qRT-PCR, and **F.** *CCR5* expression in polyI:C-stimulated THP1-Mφs transfected with *TLR3* siRNA. *; P<0.05 and **; P<0.01

The expression levels of diverse CCRs in THP-1 monocytes and THP1-Mφs were detected. As shown in Figure 1A, *CCR1,CCR4,CCR5,* and *CCR6* were expressed in both THP-1 monocytes and THP1-Mφs. *CCR1* expression was significantly higher in THP1-Mφs than in THP-1 monocytes (P=0.031). *CCR2, CCR7*, and *CXCR4* expressions at the mRNA level were not detected in THP-1 monocytes and THP1-Mφs (Fig .1A). Then, the effects of polyI:C on *CCR1, CCR4, CCR5,* and *CCR6* expressions were evaluated in THP-1 monocytes and THP1-Mφs. qRTPCR demonstrated that CCR5 expression was significantly elevated by polyI:C treatment in THP1-Mφs, while CCR5 expression was not significantly changed by polyI:C treatment in THP-1 monocytes (Fig .1B). The remarkably increased *CCR5* expression in polyI:C-stimulated THP1- Mφs was also confirmed by flow cytometry (45.9% vs. 20.8%, P=0.017, Fig .1D).

Since macrophages can recognize polyI:C stimulation through *TLR3* signalling. The effects of *TLR3* silencing on *CCR5* expression were detected in polyI:C-stimulated THP1-Mφs. Flow cytometry and qRT-PCR showed that *TLR3* siRNA transfection significantly inhibited *TLR3* expression in polyI:C-stimulated THP1-Mφs (80.2% vs. 48.8%, P=0.011, Fig .1C, E). *CCR5* expression was significantly inhibited by *TLR3* siRNA transfection in polyI:C-stimulated THP1-Mφs (P=0.044, Fig .1F).

### Polyinosinic:polycytidylic acid promoted THP-1- derived macrophage chemotaxis toward chemokine ligand 3 through toll-like receptor 3 signalling

**Fig 2:**
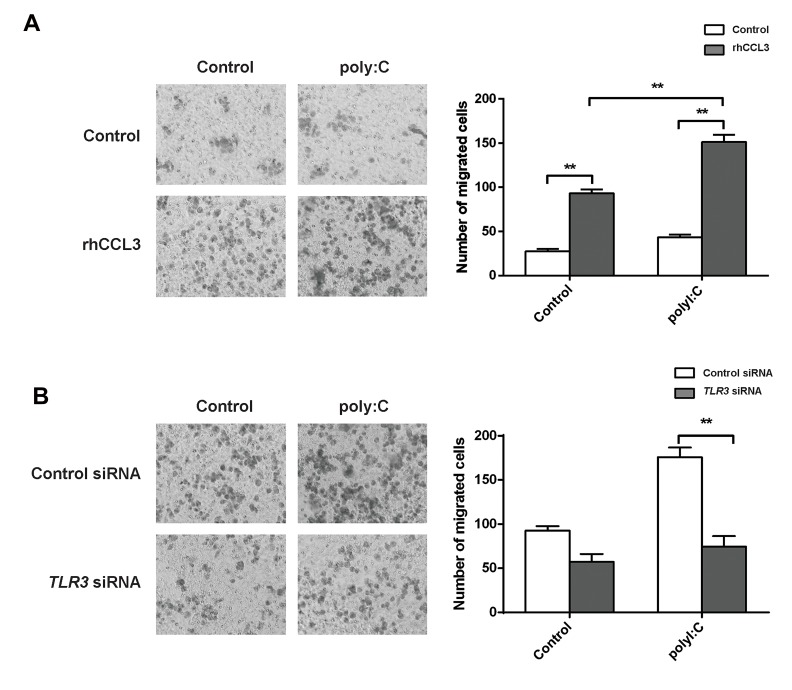
Polyinosinic:polycytidylic acid (PolyI:C) promoted THP-1-derived macrophage (THP1-Mφ) chemotaxis to chemokine ligand 3 (*CCL3*) via toll-like receptor 3 (*TLR3*) signalling. **A.** THP1-Mφs migration toward *CCL3* by polyI:C treatment and **B.** PolyI:C-stimulated THP1-Mφ migration toward *CCL3* by TLR3 siRNA transfection. **; P<0.01.

Since *CCR5* can be activated by *CCL3*, THP1-Mφ chemotaxis toward *CCL3* was analysed. As shown in Figure 2A, THP1-Mφs easily migrated to rhCCL3 (P=0.0005). PolyI:C significantly increased THP1-Mφ chemotaxis toward rhCCL3 (P=0.0006, Fig .2A). In addition, *TLR3* siRNA transfection significantly inhibited polyI:C-stimulated THP1-Mφ chemotaxis toward rhCCL3 (P=0.0029, Fig .2B).

### Polyinosinic:polycytidylic acid upregulated Jumonji domain-containing protein 1A and JMJD1C in THP- 1-derived macrophages

**Fig 3:**
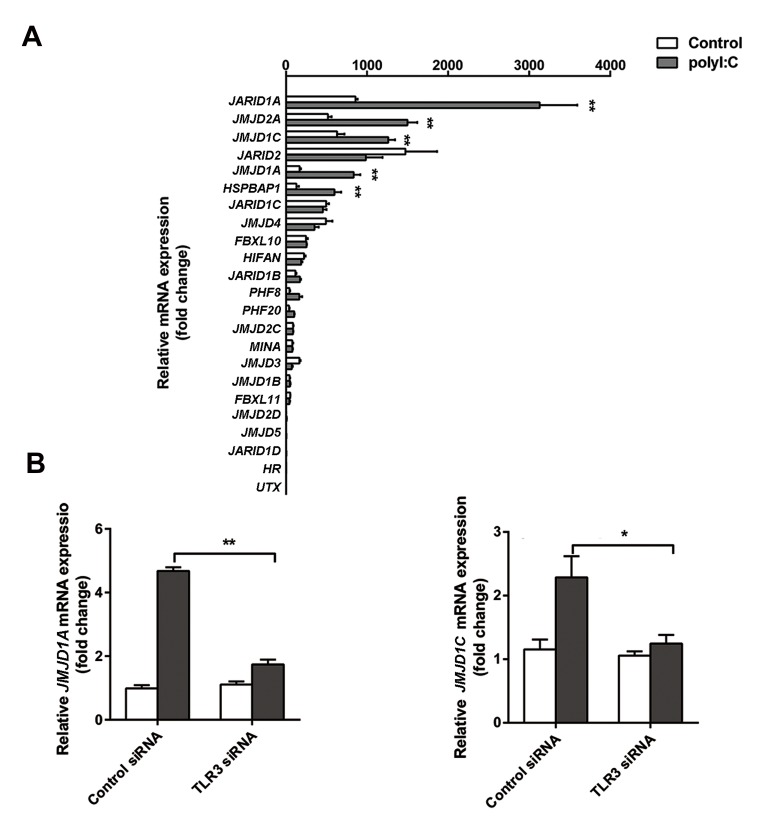
Jumonji C domain-containing histone demethylase (JHDM) family members expression in polyinosinic:polycytidylic acid (polyI:C)-stimulated THP-1- derived macrophages (THP1-Mφs). **A.** The expression levels of 23 JHDM family members in polyI:C-stimulated THP1-Mφs by quantitative real-time reverse transcriptase polymerase chain reaction (qRT-PCR,fold change at mRNA level) and **B.** Jumonji domain-containing protein (*JMJD*)1A and *JMJD1C* expression in polyI:C-stimulated THP1-Mφs transfected with toll-like receptor 3 (*TLR3*) siRNA. *; P<0.05 and **; P<0.01.

Since histone methylation is involved in the inflammatory response of macrophages, the expression levels of 23 JHDM family members were observed in polyI:C-stimulated THP1-Mφs by qRTPCR. As shown in Figure 3A, polyI:C significantly increased *JMJD1A, JMJD1C, JMJD2A, JARID1A,* and *HSPBAP1* expressions in THP1-Mφs (all P<0.01, Fig .3A). Notably, two JHDM2 subgroup members, *JMJD1A* and *JMJD1C*, were highly expressed and abundant in polyI:C-stimulated THP1-Mφs. In addition, *TLR3* siRNA transfection significantly reversed the upregulatory effect of polyI:C on JMJD1A and JMJD1C on THP1-Mφs (*JMJD1A*, P=0.002; *JMJD1C*, P=0.018, Fig .3B). Therefore, *JMJD1A* and *JMJD1C* were chosen as the targets for the following investigative processes.

### Polyinosinic:polycytidylic acid-mediated Jumonji domain-containing protein 1A upregulated chemokine receptor 5 by inhibiting H3K9me2

**Fig 4:**
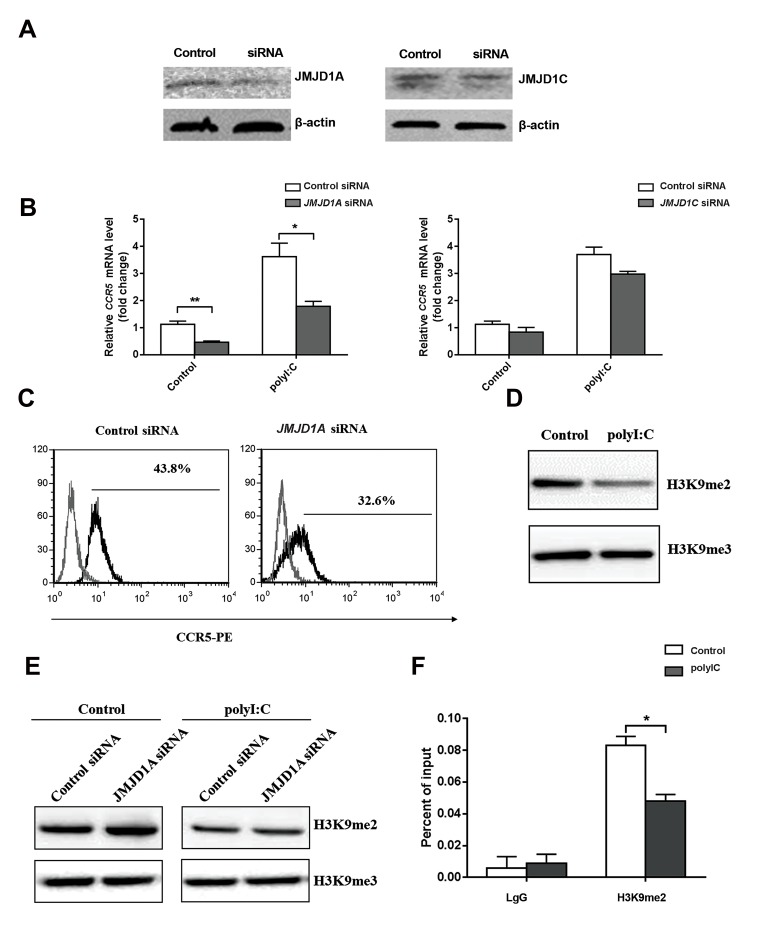
Polyinosinic:polycytidylic acid (PolyI:C)-mediated Jumonji domain-containing protein 1A (*JMJD1A*) upregulated chemokine receptor 5 (CCR5) by reducing H3K9me2. **A.** JMJD1A and JMJD1C expression in THP-1-derived macrophages (THP1-Mφs) treated with *JMJD1A* or *JMJD1C* siRNA by Western blot,** B.** CCR5 expression in polyI:C-stimulated THP1-Mφs transfected with JMJD1A siRNA and JMJD1C siRNA by quantitative real-time reverse transcriptase polymerase chain reaction (qRT-PCR) (fold change at the mRNA level), **C.** CCR5 expression in polyI:C-stimulated THP1-Mφs transfected with *JMJD1A* siRNA by flow cytometry, **D.** H3K9me2 and H3K9me3 expression in polyI:C-stimulated THP1-Mφs by Western blot (protein level), **E.** H3K9me2 and H3K9me3 expressions in polyI:C-stimulated THP1-Mφs transfected with *JMJD1A* siRNA by Western blot (protein level), and **F.** H3K9me2 expression in the promoter region of *CCR5* in THP1-Mφs by chromatin immunoprecipitation (ChIP) analysis. *; P<0.05 and **; P<0.01.

In order to investigate whether the promoted expression of *JMJD1A* and *JMJD1C* is involved in the regulation of *CCR5* expression, *JMJD1A* and *JMJD1C* were silenced in THP1-Mφs. As shown in Figure 4A, the protein expressions of JMJD1A and JMJD1C were significantly reduced in THP1-Mφs with JMJD1A or JMJD1C siRNA transfection. In addition, JMJD1A siRNA transfection significantly decreased CCR5 expression in both THP1-Mφs (P=0.007, Fig .4B) and polyI:C-stimulated THP1-Mφs (P=0.013, Fig .4B). However, CCR5 expression was not significantly influenced by JMJD1C siRNA transfection (Fig .4B). The downregulation of CCR5 expression induced by JMJD1A siRNA was also confirmed in polyI:Cstimulated THP1-Mφs by flow cytometry (43.8 vs. 32.6%, P<0.05, Fig .4C).

Since H3K9 is known to be the substrate of JMJD1A, we sought to determine if the regulatory role of JMJD1A in *CCR5* expression was dependent on H3K9 methylation. As shown in Figure 4D, H3K9me2 expression was decreased in polyI:C-treated THP1- Mφs, while H3K9me3 expression was not significantly changed. In addition, H3K9me2 was significantly upregulated by *JMJD1A* siRNA transfection in THP1-Mφs. However, H3K9me3 expression was not influenced by *JMJD1A* siRNA transfection in polyI:Cstimulated THP1-Mφs (Fig .4E). In addition, polyI:C treatment downregulated H3K9me2 expression in the promoter region of *CCR5* in THP1-Mφs (Fig .4F).

## Discussion

Macrophage chemotaxis is an important component of ALI pathogenesis. It is known that viral infections can induce alveolar macrophage recruitment, but the regulatory mechanisms of viral infection (polyI:C) on monocyte-derived macrophages are still unclear. Thus, in this study, we have explored the regulatory mechanisms of polyI:C on THP1-Mφs. The results showed that polyI:C significantly upregulated *CCR5* in THP1-Mφs and promoted THP1-Mφ chemotaxis toward *CCL3* via *TLR3* signalling. In addition, polyI:C-upregulated CCR5 was mediated by *JMJD1A*, and H3K9me2 was downregulated in the promoter region of *CCR5* in THP1-Mφs.

Since CCRs are important in macrophage chemotaxis, the expression levels of diverse CCRs were examined in THP1- Mφs after polyI:C treatment. Our results demonstrated that only *CCR5* was significantly upregulated by polyI:C treatment in THP1-Mφs. *CCR5* is a cell surface G proteincoupled receptor that is involved in inflammatory response via interaction with specific chemokine ligands, including *CCL3, CCL4,* and *CCL5* (14-16). The activation of *CCR5* and *CCL5* is required to prevent the apoptosis of virusinfected macrophages (17). In addition, *CCR5* is involved in obesity-induced adipose tissue inflammation via regulation of macrophage recruitment (18, 19). Moreover, it has been reported that polyI:C-treated macrophages can promote *CCR5* expression (20), which is consistent with the findings of our study. It was supposed that *CCR5* is involved in polyI:C-induced inflammation in THP1-Mφs. Subsequently, THP1-Mφ chemotaxis toward *CCL3* (a ligand of CCR5) was investigated. The results suggest that polyI:C significantly increased THP1-Mφ chemotaxis toward *CCL3*. A previous study reported that *CCL3* expression was significantly elevated in the lung of a murine model of LPS-induced ALI and mediated an enhanced inflammatory injury-possibly by recruiting macrophages (21). Therefore, polyI:C-upregulated *CCR5* contributes to the promotion of macrophage chemotaxis by interacting with *CCL3*.

Moreover, our results also suggest that *TLR3* siRNA transfection significantly suppressed *CCR5* expression in polyI:C-stimulated THP1-Mφs and inhibited chemotaxis toward *CCL3. TLR-3* is responsible for anti-viral immunity against several virus infections via double-stranded RNA recognition and the activation of multiple antiviral factors in macrophages (20). Similarly, *TLR-3* is activated in macrophages in response to encephalomyocarditis infection via type 1 IFN production. It has been reported that *CCR5* may participate in virus replication and acts as the primary receptor for regulating encephalomyocarditis infection in mediating inflammatory response–related genes in macrophages (22). These results indicate that macrophages may recognize polyI:C stimulation through TLR3 signalling. PolyI:C may upregulate *CCR5* expression and promote THP1-Mφ chemotaxis toward *CCL3* through *TLR3* signalling.

Histone demethylation, dynamically regulated by JHDMs, is implicated in the regulation of inflammatory response of macrophages (23). Previous studies have reported that *JMJD3* is over-expressed in LPS-activated macrophages, which regulates diverse genes involved in LPS-induced immune and inflammatory responses (10, 24). However, few studies have focused on the regulatory mechanisms of polyI:C in histone demethylation in macrophages. In this study, the expression levels of 23 JHDM family members were detected in polyI:Cstimulated THP1-Mφs. The expression levels of *JMJD1A, JMJD1C, JMJD2A, JARID1A,* and *HSPBAP1* were significantly increased by polyI:C in THP1-Mφs, while that of *JMJD3* was not significantly changed. These results indicated that the effects of polyI:C on inflammatory responses of macrophages might differ from LPS. Since *JMJD1A* and *JMJD1C* could be regulated by *TLR3* in polyI:C-stimulated THP1-Mφs, the regulatory roles of *JMJD1A* and *JMJD1C* on *CCR5* were further analysed in this study. It was revealed that *CCR5* was significantly downregulated by *JMJD1A* siRNA transfection in polyI:Cstimulated THP1-Mφs, while *CCR5* expression was not significantly influenced by *JMJD1C* siRNA transfection. The regulatory role of *JMJD1A* has been found to affect the proliferation, migration, and invasion of cancer cells in various cancer types (25-27). It has been reported that *JMJD1A* inhibition suppresses tumour growth by downregulating angiogenesis and macrophage infiltration (28). Our findings indicate that polyI:C treatment may induce a similar macrophage inflammatory response with cancer; PolyI:C may enhance *CCR5* expression by upregulating *JMJD1A* in THP1-Mφs.

Since *JMJD1A* is a H3K9 demethylase, the H3K9 methylation state of *CCR5* was analysed in polyI:Cstimulated THP1-Mφs. Our results showed that H3K9me2 expression was significantly decreased by polyI:C treatment in THP1-Mφs. H3K9me2 downregulation might have attributed to the upregulation of *JMJD1A*. However, H3K9me3 expression was not significantly influenced by polyI:C treatment. Our findings indicate that the regulatory role of *JMJD1A* on *CCR5* was dependent on H3K9me2. In addition, H3K9me2 was upregulated by *JMJD1A* siRNA transfection in THP1-Mφs, while H3K9me2 expression was not significantly influenced by JMJD1A siRNA in polyI:C-stimulated THP1-Mφs. This may be explained by the fact that some other upregulated JHDMs induced by polyI:C, such as *JMJD1C*, and *JMJD2A* may share a target with *JMJD1A. JMJD1C* and *JMJD2A* exhibit redundant effects on H3K9me2 expression. The presence of H3K9me2 in the promoter region of target genes typically results in reduced expressions of its targets. A previous study has reported that H3K9 exhibits a low methylation level in response to the activation of dendritic cells and is erased from the promoters of some activated inflammatory genes (29). Consistent with the results of that study, our results reveal that H3K9me2 expression was significantly reduced by polyI:C treatment in the promoter region of *CCR5* in THP1-Mφs. We suspected that polyI:C-mediated *JMJD1A* upregulation may upregulate *CCR5* by reducing H3K9me2 in the promoter region of *CCR5*. Interestingly, *JMJD1A* is also a hypoxiainducible gene that has been found to be upregulated in hypoxia-stimulated macrophages. However, hypoxia treatment decreases *CCR5* expression via H3K9me2 upregulation in the promoter region of *CCR5* (30). This may be explained by the effects of hypoxia-induced repressive JMJDs, which can overwhelm the effects of *JMJD1A*.

## Conclusion

The present study revealed that polyI:C upregulated *JMJD1A* expression in THP1-Mφs, thereby elevating the CCR5 expression by reducing H3K9me2 in the promoter region of *CCR5* via *TLR3* signalling. However, this study is still limited to the cellular level, and the validation of these results in animal models is required in future research.
